# Highly Efficient Generation of Germline Mutations Using CRISPR/Cas9 in the Speckled Wood Butterfly *Pararge aegeria*

**DOI:** 10.1002/ece3.71624

**Published:** 2025-06-29

**Authors:** Anna B. Shoshan, Kalle Tunström, Christopher W. Wheat, Karl Gotthard

**Affiliations:** ^1^ Department of Zoology Stockholm University Stockholm Sweden; ^2^ Bolin Centre for Climate Research Stockholm University Stockholm Sweden; ^3^ Department of Biology Lund University Lund Sweden

## Abstract

To date, the use of CRISPR/Cas9 technology in ecological‐model species for validating genotype to phenotype connections has focused primarily on visual phenotypes using G_0_ mutations, coupled with analyses of resulting mosaic phenotypes. However, studies of physiological phenotypes necessitate germline mutations in order to assess non‐visible phenotypic effects, and thus, dedicated efforts to develop efficient germline mutations in ecological model species are needed. Here, we applied the CRISPR/Cas9 technology to an ecological model species, the speckled wood butterfly (*Pararge aegeria*). We targeted *yellow‐y*, which is required for the production of black melanin, as *yellow‐y* loss of function (LOF) mutations are not lethal and easy to phenotype, affording efficient assessment of G_0_ and germline mutations. To explore what factors may affect the efficiency of transformation, we employed four alternative treatments, including variation in sgRNAs and their concentrations. Color changes in the head capsule of first larval instars, as well as adult wing color, were used as indicators of successful knockouts. Individuals with wings that were at least 50% transformed were mated, with their G1 offspring assessed for the presence of germline mutations. Our CRISPR/Cas9 technique was highly efficient at generating LOF mutations in *yellow‐y*. Across all treatments, nearly 80% of adults exhibited mosaic LOF phenotypes, of which nearly 30% appeared to have 100% LOF phenotypes. Crosses between adults exhibiting at least 50% LOF phenotypes resulted in fully transformed offspring, revealing a high incidence of germline LOF mutations in *yellow‐y*. We provide a detailed protocol on how to obtain high germline LOF mutation efficiency in order to advance the study of genotype–phenotype connections for non‐visible physiological traits across natural populations of this and other model ecological species.

## Graphical abstract

We demonstrate the efficient use of CRISPR/Cas9 to generate germline loss‐of‐function (LOF) mutations in the speckled wood butterfly (*Pararge aegeria*), an ecological model species. Targeting the *yellow‐y* gene, we achieved nearly 80% mosaic LOF phenotypes in adults and high germline mutation rates in offspring. Our protocol provides a foundation for studying genotype–phenotype connections, including non‐visible physiological traits, in ecological model organisms.

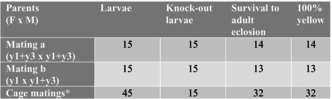

## 1 Introduction

The field of ecology and evolution is now routinely harnessing the power of population and comparative genomic analyses to gain insights into the genomic regions causally involved in adaptive phenotypes (Savolainen et al. 2013; Aguirre‐Liguori et al. 2021). However, connecting genotype with phenotype in ecological model species is challenging and fraught with high false‐positive rates due to a myriad of factors, such as population stratification and environmental influences (Wellenreuther and Hansson 2016; Schielzeth et al. 2018; Gudmunds et al. 2022). Thus, genomic loci identified using population genomic tools are generally best considered candidates for phenotypic effects and require functional validation before causal language is warranted. While such functional validation is common within model species communities (e.g., *Drosophila melanogaster* , *Arabidopsis thaliana* ) (Todesco et al. 2010; Curtin et al. 2017; Rohde et al. 2018; Chong and Stinchcombe 2019; Zhang et al. 2020), validation of gene to phenotype causality remains comparatively rare in ecological model species (van der Burg et al. 2020; Lindestad et al. 2024). In order to advance functional validation in ecological model species, here we present results demonstrating high‐efficiency targeted mutagenesis of a long‐used ecological model species, the speckled wood butterfly (*Pararge aegeria*).

One of the fastest evolving means of testing hypotheses of genotype to phenotype connections in ecological model species, and Lepidoptera in general, is CRISPR/Cas9 mutagenesis. CRISPR/Cas9 is a simple, cost‐effective, and efficient genetic tool for creating site‐specific mutations (Baci et al. 2022), and has increased the understanding of gene function in lepidopteran development, metamorphosis, pigmentation, as well as mating and reproduction (Li et al. 2021; Ficarrotta et al. 2022; Han et al. 2022; Okamura et al. 2022; Xu et al. 2022; Chakraborty et al. 2023; Hanly et al. 2023; Shirk et al. 2023; Tunström et al. 2023; Li, Lao, et al. 2024; Li, Li, et al. 2024; Tendolkar et al. 2024).

However, studied traits have almost exclusively been visible phenotypes, wherein successful CRISPR/Cas9 mutagenesis is easily detected among the injected individuals (generation zero, G0) as mosaics of mutant and wild type phenotypes. Assessing G0 mosaics of non‐visible physiological phenotypes is exceptionally challenging, and therefore germline mutations are needed in such cases to ensure modification across all relevant tissues in assayed individuals. For example, when studying digestive physiology in *Prieris brassicae*, Okamura et al. (2022) used CRISPR/Cas9 to study the functional role of two genes, nitrile specifier protein (NSP) and major allergen (MA). By working with verified germline knockout lines of NSP and MA, the authors were able to investigate the function as well as the fitness consequences of not having functional copies of these genes in relation to host plant chemical defenses.

Despite the necessity of germline mutations in investigating physiological non‐visible traits within lepidopterans, extensive germline work has only been performed in the domesticated silk moth *Bombyx mori* (Zhang and Reed 2017). Additionally, the induction of germline mutations using the CRISPR/Cas9 method in Lepidoptera ecological model species is still relatively rare (Shirai et al. 2021; Connahs et al. 2022; Li et al. 2022; Okamura et al. 2022; Wang et al. 2023). One reason for this is that the creation of germline mutations can be challenging, as it may require a relatively high transformation efficiency to reach individuals with germline mutations. Additionally, the optimal Cas9‐sgRNA concentration and number of sgRNAs per treatment may vary between different genes and systems (Bassett et al. 2013; Cong et al. 2013; Pattanayak et al. 2013; Wang et al. 2013; Mohr et al. 2016; Liu, Han, et al. 2020; Liu, Zhang, et al. 2020), meaning that a protocol for creating mutant lines in *B. mori* (Brady et al. 2020) might not be as efficient when applying CRISPR/Cas9 to a different system, which renders protocol optimization necessary. However, we note that a recent publication reported high efficiency CRISPR/Cas9 germline mutations in the crop pest *Ostrinia nubilalis* , where the authors present in excellent detail their work on generating loss of function mutations in this moth for three different clock genes (Dayton et al. 2024).

To measure the mutation efficiency of CRISPR/Cas9, several studies have utilized the creation of visible phenotypic changes and created knock‐outs of the *yellow‐y* gene, a melanin‐promoting factor (Perry et al. 2016; Wang et al. 2021). The *yellow‐y* protein is necessary for the production of black melanin in the cuticle (body and wings) of many adult insects, as well as in the pupa, larvae, and larval head capsule in some species (Futahashi and Fujiwara 2007; Futahashi and Osanai‐Futahashi 2021; Shirai et al. 2021; Wang et al. 2021). By targeting a pigment‐influencing gene, mutations induced in the microinjected generation (G0) will be visually apparent, and the proportion of transformed individuals can be evaluated. CRISPR/Cas9 mutations in the *yellow‐y* gene have been generated for several Lepidoptera (Perry et al. 2016; Liu, Han, et al. 2020; Liu, Zhang, et al. 2020), documenting that the *yellow‐y* gene is necessary for the dark brown background color, such as that found in the adult wings of *P. aegeria* (Livraghi, Martin, et al. 2018). Across many Lepidoptera, first instar larvae additionally have dark brown head capsules, which are likely influenced by the *yellow‐y* gene. Besides influencing pigmentation, the *yellow‐y* gene has been shown to influence hatching success, larval segmentation, and molting in *Spodoptera litura* (Liu, Han, et al. 2020; Liu, Zhang, et al. 2020; Shirai et al. 2021) as well as male courtship behavior in *D. melanogaster* and the butterfly *Bicyclus anynana* (Drapeau et al. 2003; Connahs et al. 2022). Therefore, we expect diverse pleiotropic effects from knocking out the *yellow‐y* gene in Lepidoptera.

Here, we investigated the mutation efficiency of CRISPR/Cas9 in the butterfly *P. aegeria* using mutations in the *yellow‐y* gene and assessed the resulting color differences of the first instar head capsule and adult wings. While previous work used CRISPR/Cas9 to generate G0 mosaics in *P. aegeria* to assess this gene's role in wing pigmentation (Livraghi, Martin, et al. 2018), that study provided neither quantitative details on their methods nor the penetrance of their CRISPR/Cas9 mutations. Here, we created four treatments varying in the concentration of Cas9‐sgRNA in the complex, the utilized sgRNAs, and the number of sgRNAs per complex to detect potential differences in mutation efficiency depending on methodology. Importantly, we wanted to test whether we could induce germline mutations that would allow us to produce a second generation carrying the target mutation by breeding individuals of the microinjected generation showing more than 50% color transformation. Additionally, we investigated potential side effects of microinjections and pleiotropic effects of the *yellow‐y* gene on different life history traits.

## 2 Methods

### 2.1 The Study Species

**FIGURE 1:**
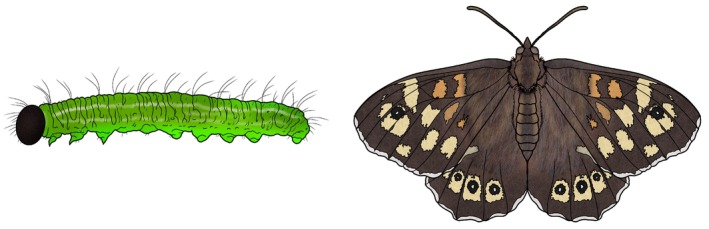
As a first instar, the larva has a dark brown head capsule. The adult has a brown background color, yellow and/or orange patches, and eye spots on both fore‐ and hind wings. The adult background color is influenced by a knock‐out of the *yellow‐y* gene (Livraghi, Martin, et al. 2018). The same may be the case for the first instar larval head capsule.

The speckled wood butterfly (*P. aegeria*) is a Palearctic woodland butterfly (Livraghi, Vodă, et al. 2018) with a variety of grass species as host plants, including *Dactylis glomerata* and *Poa annua* (Shreeve 1986). Newly hatched first instar larvae are white with a dark brown head capsule, but after feeding on the host plant, the white body becomes green, making the larvae very cryptic on the host (Figure 1). As the larva molts into the second instar, it drops the dark head capsule, and the head and body are green throughout the remaining 3 larval instars (4 in total). The pupa can be either green or brown and is well camouflaged in the natural environment (Van Dyck et al. 1998). In Sweden and most of Europe, the adult has a dark brown background color, yellow and orange patches, and eye‐spots on both the fore‐ and hind wings (Figure 1) (Packer 1984; Van Dyck, Matthysen, et al. 1997; Van Dyck et al. 1998; Talloen et al. 2009). Adult morphology can vary slightly in how pale the background color is, the size of the patches, and the number of eye‐spots depending on factors like the time of the season, sex, population, and larval food stress (Packer 1984; Van Dyck, Matthysen, et al. 1997; Windig et al. 2004; Talloen et al. 2009; Taylor‐Cox et al. 2020). In both sexes, the background color and size of patches are intermediate to highly genetically determined (Van Dyck et al. 1998). In the present study, we hypothesize that the dark brown head capsule of the first instar larva necessitates *yellow‐y* gene function, as has been shown for the adult background color (Livraghi, Martin, et al. 2018), and that a knock‐out of this gene will cause significant color changes in both life stages. The pupal color was not observed, as the larvae were kept on fresh green grass, which should result in mainly green pupae (Van Dyck et al. 1998). The green color is not expected to be influenced by the *yellow‐y* gene.

#### 2.1.1 Identification of the *yellow‐y* gene

We downloaded the reference genome and annotation data from NCBI. The annotation was generated by ENSAMBL, as is detailed in the genome report by Lohse et al. (2021). Using these resources, we determined the cDNA sequence of the *yellow‐y* gene in *P. aegeria*. The *yellow‐y* gene has the gene ID ENSPAGG00005015484 and is located on chromosome 5 at position 7,853,744–7,868,807. In addition to the provided annotation, a validation of the gene was conducted using the Basic Local Alignment Search Tool (BLAST) (Camacho et al. 2009), comparing it with the orthologs the painted lady butterfly ( *Vanessa cardui* ) (GCA_022405095.1; Zhang et al. 2021) and the mycalesine butterfly (*Bicyclus anynana*) (GCA_900239965.1; Nowell et al. 2017). Notably, in both these species, researchers have previously achieved successful G_0_ mosaic knock‐out of the *yellow‐y* gene (Perry et al. 2016; Matsuoka and Monteiro 2018). The risk of affecting potential off‐target sites was reduced by examining the *P. aegeria* reference genome and the two orthologs for duplicates or closely related genes. The same procedures were performed for the *SPR* gene, which was included in this study in combination with a separate experiment. SPR is an enzyme that catalyzes reactions in the pteridine biosynthesis pathway (Descimon 1975) and is responsible for pigmentation across a range of taxa, including butterflies and lizards (Andrade and Carneiro 2021; Futahashi and Osanai‐Futahashi 2021). In butterflies, the use of pteridines as wing pigments is largely restricted to pierid species; however, many species use pteridines for eye pigmentation. Consequently, *SPR* LOF mutations may influence pigmentation in the compound eye of *P. aegeria*, an aspect not affected by *yellow‐y* in this species (Livraghi, Martin, et al. 2018). The *SPR* gene (LOC_120630788) is located on chromosome 17, and was validated using the same approach applied for the *yellow‐y* gene: using BLAST, existing annotations, and sequences from an ortholog in the domestic silk moth ( *Bombyx mori* ) (SPR = C0STP5).

### 2.2 Design of sgRNAs

**FIGURE 2:**
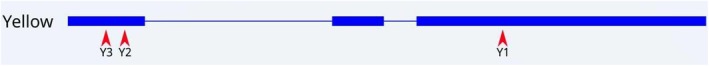
Target locations of the sgRNAs y1, y2, and y3 in the *yellow‐y* gene. The blue blocks are exons, and the target sites are marked with red arrows. The gene is visualized in the 5′ to 3′ direction.

The sgRNA target sequences were identified and generated manually following the protocol outlined by Perry et al. (2016). The process involved several steps, beginning with the manual identification of PAM‐sites (NGG sequences) within exons. Recognition and binding of the Cas9 to the targeted DNA sequence rely on the presence of a PAM‐site (Sternberg et al. 2014). In the next step, we identified which among these PAM‐sites had flanking sequences with GC‐ratios ranging from 40% to 60%. Finally, we checked for potential off‐target sequences by blasting the potential sgRNAs against the *P. aegeria* reference genome using NCBI BlastN (with the BlastN‐short flag and an e‐value filter set at 0.01). Any candidate sgRNA sequence was discarded if it had 15 or more bp in alignment somewhere else in the genome. Three distinct sgRNA target sequences for the *yellow‐y* gene were selected (Table 1 and Figure 2), along with a single sgRNA target sequence targeting the first exon of the *SPR* gene (Table 1).

**TABLE 1:** Names of sgRNAs used in this experiment and their target sequences (the PAM sites are underlined).

Name of sgRNA	Target gene	Target sequence
Pae_y_1	*Yellow‐y*	CGGAGACTTTAACATAGCTGG
Pae_y_2	*Yellow‐y*	GCAAAACGCTTTACCTGTTGG
Pae_y_3	*Yellow‐y*	ACAACAGGCCCTCCAGACTGG
Pae_s_4	*SPR*	ATCTATCTACTGCATCCGAGG

### 2.3 Generating sgRNAs

Each sgRNA target sequence (excluding the PAM‐site) was combined with the remaining structure of an sgRNA sequence and then synthesized into DNA fragments at IDT (Coralville, Iowa, USA). These guide sequences additionally contained two primer binding sites (P505 and M13f), flanking the sgRNA sequence to facilitate DNA amplification through PCR, as well as a T7‐promoter sequence to allow the transcription of the DNA sequence into RNA. In sum, the guide sequence comprised the following elements: an M13F sequence, a T7‐promoter sequence, a spacer sequence, the target sequence, a Cas9 binding site, and a P505 sequence.

PCR amplification of the guide sequences was conducted employing the primers M13f (GTAAAACGACGGCCAG) and P505 (AAAAAAAAGCACCGACTCGGTGCC), along with Platinum Taq (Invitrogen, cat. 10,966–034). The thermal cycling program was: 94°C × 120s, 35 cycles of “94°C × 30s, 62°C × 30s, 72°C × 30s” followed by 72°C × 4min. For each sgRNA, a total of four 50 μL PCR reactions were run and subsequently combined. The combined PCR products were purified using the MinElute PCR Purification Kit with a MinElute spin column (cat. 28004, Venlo, Netherlands), following the manufacturer's protocol. The resulting templates were then transcribed into RNA to generate the sgRNAs, adhering to the protocol provided in the Lucigen AmpliScribe T7‐Flash Transcription kit from Epicenter/Illumina (cat. ASF3507, Madison, WI, USA). Lastly, the transcribed sgRNAs were purified using the previously mentioned purification kit. The resulting products were resuspended in Qiagen buffer EB, quantified using Qubit, and subsequently diluted to a concentration of 1000 ng/μL before being stored at −20°C until use. In preparation for injection, the sgRNA(s) were combined with Cas9‐NLS protein (Cat. no. CP01‐50, PNA Bio, Newbury Park, CA, USA), which had been resuspended in nuclease‐free water, at a 1:1 ratio. The complex was then diluted with nuclease‐free water to its final concentration. Each day of the experiment, one of the given treatments was utilized for injections, over a span of 4 consecutive days. The treatment complex was kept on ice throughout the day of injections.

### 2.4 Treatments

To test for the most effective solution, we created four different treatments of Cas9‐sgRNA complexes that varied in sgRNA(s) as well as the concentration of Cas9‐sgRNA:

1. y1 + y3 (250 + 250 ng/μL), Cas9 (500 ng/μL)
2. y1 + s4 (125 + 125 ng/μL), Cas9 (250 ng/μL)
3. y1 (500 ng/μL), Cas9 (500 ng/μL)
4. y2 (250 ng/μL), Cas9 (250 ng/μL)

The “s4” sgRNA was chosen as a secondary target site in combination with a separate experiment, investigating the possible function of *SPR* on compound eye pigmentation in this species. *SPR* LOF is not expected to cause lethal mutations, or affect the same tissues as the *yellow‐y* gene (Livraghi, Martin, et al. 2018; Andrade and Carneiro 2021).

### 2.5 Collection and Breeding of *P. aegeria*

In June 2021, wild‐mated females of *P. aegeria* were collected from the Baltic Island of Gotland (57.40 N, 18.52 E, 15 females). The F1 offspring were raised in cycles of 22 h light:2 h darkness, and 23°C, to induce non‐diapause development, while the F2 generation was reared under a set of daylengths (shorter than 18.6 h light), and 17°C–18°C, which is known to induce pupal diapause in this population. The diapausing pupae were subsequently moved to a 2°C climate cabinet, with constant darkness, to simulate winter. In February 2022, after diapausing for 139–167 days, 104 pupae were moved to a climate room (12 h light:12 h darkness, 17°C), to induce adult eclosion. This resulted in 92 adults that could be used to produce F3 offspring. In total, 35 independent matings were successfully completed, after which the mated females were placed individually in 0.5 L plastic cups with *Poa annua* grass, for oviposition. At all times, the females had access to sugar water on a piece of cotton. The oviposition room had a cycle of 8 h light:16 h darkness (light period temperature: 27°C, dark period temperature: 18°C).

### 2.6 Egg Preparation

To ensure a short time until injection of the eggs, each female's cup was exchanged for a new cup with *Poa annua* grass every 2 h. The cups containing eggs on *P. annua* were immediately brought to a lab, where the eggs were placed on double‐sided adhesive tape on ethanol‐cleaned microscope slides. When placing the eggs on the double‐sided tape, they were preferably still on the *P. annua* grass, but if that was not possible because the eggs fell off the host plant, they were placed directly on the tape. Afterwards, an effort was made to cover the remaining tape as completely as possible with *P. annua* leaves, to keep the larvae from getting stuck after hatching. Each slide was marked with the slide number, treatment, and date of injection.

### 2.7 Injection

The slide with eggs was placed under the microscope, and the eggs were injected with the sgRNA/Cas9 complex, using a thin glass needle created from borosilicate glass capillaries (WPI, Item number 1B100‐4, 1/0.58 mm OD/ID, length 4 in. [100 mm]), and a Narishige PC‐10 Dual Stage Glass Micropipette Puller. After injection, the maximum time until injection (from the time the female was given the cup with grass to when the injection was done for a full slide), as well as the number of eggs on the slide, were noted. The slides with injected eggs were then placed in a petri dish next to a piece of paper towel with distilled water, to keep the humidity high. The petri dish was sealed with parafilm and placed in a climate room with a 20 h light:4 h dark cycle and 23°C. A total of 54 slides were created, with between 1 and 22 eggs in total, and the maximum injection time varied from 2 h and 20 min to 5 h and 10 min. In total, 555 eggs were injected (Table 2). As controls, 34 eggs remained on the grass in the original cup from their mother (non‐injected control). In addition, we added a control treatment, where 114 eggs were injected with nuclease‐free water (water control), to address if microinjection affects survival (Table 2). We used water, as it was the main ingredient in the treatment complexes. To mimic the CRISPR/Cas9 treatments, water was added to an Eppendorf tube and placed on ice before use. The non‐injected control and water control groups are referred to as Generation 1 (F0) throughout the text, to make it clear that they belong to the same generation as the individuals injected with a sgRNA/Cas9 complex (G0).

**TABLE 2:** The survival of larvae and adults, and how they were scored in each phenotypic category in each CRISPR/Cas9 treatment. In brackets are the following: Egg survival (as a proportion of eggs injected), knock‐out larvae (as a proportion of egg survival), adult eclosion (as a proportion of egg survival), and adult phenotypic category (as a proportion of individuals phenotyped within each treatment).

Treatment	Concentration (ng/μL)	Eggs injected/started	Egg survival	Knock‐out larvae	Adult eclosion	Wild‐type	< 50% yellow	> 50% yellow	100% yellow
y1 + y3/Cas9	250 + 250/500	137	45 (0.33)	35 (0.78)	27 (0.60)	3 (0.11)	2 (0.07)	5 (0.19)	17 (0.63)
y1 + s4/Cas9	125 + 125/250	263	54 (0.21)	25 (0.46)	33 (0.61)	7 (0.23)	10 (0.32)	11 (0.35)	3 (0.10)
y1/Cas9	500/500	57	21 (0.37)	14 (0.67)	13 (0.62)	3 (0.23)	2 (0.15)	5 (0.38)	3 (0.23)
y2/Cas9	250/250	98	26 (0.27)	9 (0.35)	17 (0.65)	5 (0.31)	3 (0.19)	7 (0.44)	1 (0.06)
Water Control		114	45 (0.39)	45 (0.39)	14^a^ (0.78)				
Non‐injected Control		34	32 (0.94)	32 (0.94)	14^a^ (0.78)				

^a^ Only 18 larvae from each of the control treatments were followed to adult eclosion.

### 2.8 After Injection

Every day, the slides were checked for hatchings. As soon as one egg hatched on a slide, the larva was scored as being a knock‐out or not, depending on the head capsule appeared black/dark brown, or light brown. The open containers had distilled water added to the paper towel daily, to keep humidity high inside. The hatched larvae were placed in plastic cups, with 1–3 individuals per cup, on *Poa annua* grass, and fed ad libitum until pupation. The cups were checked for pupation regularly. Pupation day was noted, and the pupae were given 1–2 days to harden, after which they were weighed, and placed individually in 0.2 L plastic containers. After eclosion and release of meconium, the adults were weighed on a Precisa 205 A SCS balance (precision 0.1 mg). The color of each adult was scored, and assigned to one of four categories: “wildtype,” if the background color was brown as the wildtype; “100% yellow,” if the background color appeared fully yellow; “> 50% yellow” or “< 50% yellow,” when an individual showed a mosaic of colors on its wings. The background color of both the dorsal and ventral side of the wings was assessed. One adult from the non‐injected control treatment was scored as more than 50% yellow. We assume that this individual was misplaced, and removed it from all analyses. Three larvae from each yellow treatment, that were scored as knock‐outs based on their head capsules, were followed until adult eclosion, to assess any correlation between knock‐out head capsules and adult morphology.

### 2.9 Assessment of Germline Mutations

Approximately 25 adults belonging to the two categories with the highest penetrance: “> 50% yellow” (at least 1 female and 6 males), and “100% yellow” (at least 7 females and 8 males) were marked with their ID numbers on their wings, and placed in one of two net cages (30 × 40 × 50 cm), containing *Dactylis glomerata* . The cages were maintained in a climate room at 23°C, under a 20 h light:4 h dark cycle. Adults were from treatments y1 + y3 (at least 4 females and 9 males), y1 + s4 (at least 3 females and 4 males), and y1 (at least 2 females and 1 male). Observing all matings would have required near‐constant monitoring, as this species typically mates for around 15 min. Instead, the cages were checked regularly, resulting in two observed matings: Mating 1: Females a (y1 + y3 [100% yellow]) × Male a (y1 + y3 [100% yellow]) and Mating 2: Female b (y1 [100% yellow]) × Male a (y1 + y3 [100% yellow]). *P. aegeria* females typically mate only once, while males can mate with multiple females under laboratory conditions (Velde et al. 2011). Therefore, after each mating, the male was returned to the mating cage. Mated females were removed, and placed individually in 0.5 L plastic cups with *Poa annua* grass to lay eggs. After hatching, 15 larvae from each of the two females were randomly chosen, and placed in separate cups with ad libitum *Poa annua* . During development, each individual was scored for pupation date, pupal weight, date of adult eclosion, adult weight, sex, and adult phenotype.

Unobserved matings in the mating cages also produced fertile eggs that hatched, and, in total, 45 larvae from this group were randomly chosen, scored for color of the head capsule, and placed in two 30 × 40 × 50 cm net cages with *Dactylis glomerata* (20 or 25 larvae in each of the two net cages). Each adult was phenotyped as either “wildtype,” or “yellow,” depending on whether their background color appeared dark brown, as the wildtype, or yellow due to a knock‐out of the *yellow‐y* gene.

### 2.10 Statistical Analysis

All analyses were done in R version 4.1.2, with a significance level set at 0.05 throughout. All significant effects in general linear models were further examined by Tukey tests, using the emmeans package (Lenth 2021), to identify which of the treatments differed significantly.

We first analyzed if the CRISPR/Cas9 technique targeting the *yellow‐y* gene overall had any significant effect on the morphology of the larvae or adults, separately. This was done using a chi‐square test, and comparing the frequency of “wildtype” to “yellow knock‐out” between all four *yellow‐y* treatments (treatment y1 + y3, y1 + s4, y1, and y2 pooled), and the two controls (water‐injected control and non‐injected control pooled). Individuals were considered “yellow knock‐out” if they belonged to any of the three categories showing any level of transformation; “< 50% yellow,” “> 50% yellow,” and “100% yellow.”

Secondly, we investigated if the four CRISPR/Cas9 treatments differed in their efficiency. This was done by scoring the larval and adult pupal phenotypes as either “wildtype” or “yellow knock‐out,” adhering to binomial distributions. The category “yellow knock‐out” again included individuals from all three knock‐out categories (“< 50% yellow,” “> 50% yellow,” and “100% yellow”). A generalized linear model was fitted to the data to test if the response variable “yellow knock‐out” phenotype was dependent on the factor treatment (y1 + y3, y1 + s4, y1, and y2).

Adults assessed as 100% knock‐outs were most likely to carry the mutation in the germline. Therefore, we tested if the four CRISPR/Cas9 treatments differed in the frequency of individuals appearing as fully knock‐out. This was done with a generalized linear model, as mentioned above, but where “yellow knock‐out” only included individuals from the “100% yellow” category. For this analysis, all individuals in the categories “< 50% yellow” and “> 50% yellow” were pooled with the “wildtype” category.

Microinjections, as well as injection with a sgRNA/Cas9 complex, may affect survival. Therefore, survival of eggs (hatching rate) and survival to adult eclosion (proportion of hatched larvae that eclosed as adults) were compared between the treatments. For both traits, each individual was scored as having survived or not. To test the effects on survival, we fitted a generalized linear model to the data and investigated if the response variable survival (both as egg and to adult eclosion) was dependent on the factor treatment (y1 + y3, y1 + s4, y1, y2, water‐injected control, and non‐injected control).

Knock‐out of the *yellow‐y* gene may affect survival by itself. Therefore, survival to adult eclosion was compared between the non‐injected wildtype control (F0) and the germline knock‐outs (G1^‐*yellow‐y*^). We could not test for egg survival, as we did not register egg survival in the germline knock‐outs. Each larva was scored as surviving or not to adulthood. Only the germline knock‐outs from the two observed matings were included, as they were reared in separate cups, as the control. Again, a generalized linear model was fitted to the data to test if survival differed between the control and germline knock‐outs (F0 and G1^‐*yellow‐y*^).

Knock‐out of the *yellow‐y* gene may have pleiotropic effects on other life history traits. Therefore, we performed two‐way ANOVA tests, with the response variable being one of the life history traits: larval development time, pupal development time, pupal weight, or adult weight, and the explanatory factors being the treatment (non‐injected wildtype control and germline knock‐outs), as well as sex. Again, only the germline knock‐outs from the two observed matings were included.

## 3 Results

### 3.1 Injected Generation

**FIGURE 3:**
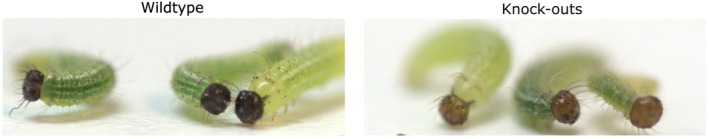
Pictures illustrating the phenotypic categories for larvae *yellow‐y* knock‐outs. The wildtype first instar head capsule is dark brown (left) and was scored as knock‐out if it appeared light brown (right).

**FIGURE 4:**
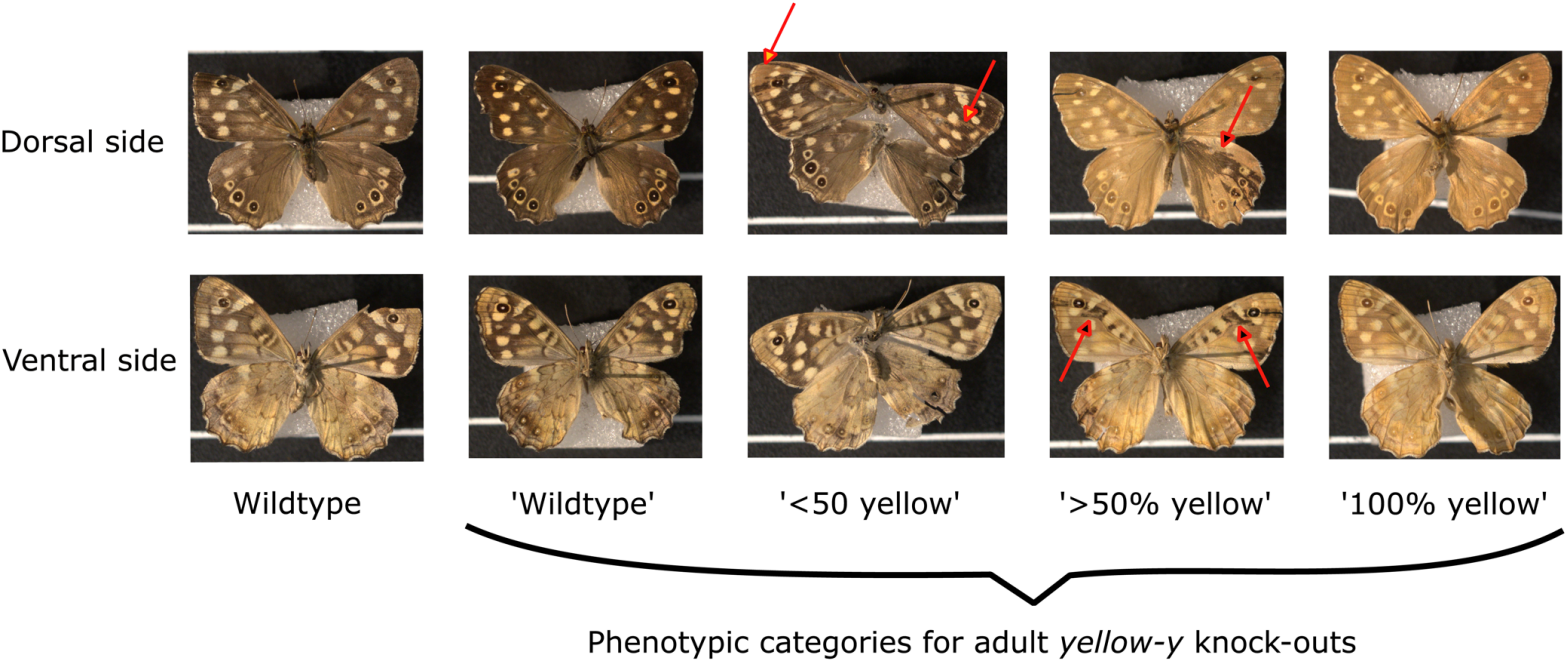
Pictures illustrating the phenotypic categories for adult *yellow‐y* knock‐outs. Five individuals are shown, with the dorsal side in the top row and the ventral side in the bottom row. The wildtype adult (left) has a dark brown background color. Knockout adults were scored as “wildtype,” “< 50% yellow,” “> 50% yellow,” or “100% yellow,” depending on the proportion of the background color that appeared yellow. Arrows indicate areas where individuals with mosaic coloration show distinct patches.

The CRISPR/Cas9 treatments had a significant effect on the phenotypes of both larvae and adults (Larvae: χ^2^_(1289)_ = 111.29, *p* < 0.001; Adults: χ^2^_(1114)_ = 50.98, *p* < 0.001). Across the four CRISPR/Cas9 treatments, 83 out of 146 larvae (56.85%) had knock‐out head capsules, 69 out of 87 adults (79.31%) had at least some cells transformed, and 24 out of 87 adults (27.59%) appeared fully transformed (Figures 3 and 4 and Table 2).

The four CRISPR/Cas9 treatments differed significantly in efficiency in regard to the proportion of larvae that had a knock‐out head capsule (χ^2^(3) = 17.14, *p* < 0.001). The y1 + y3 treatment, with a sgRNA concentration of 500 ng/μL (250 ng/μL y1 and 250 ng/μL y3) and a Cas9 concentration of 500 ng/μL, was significantly more successful than the two treatments with a sgRNA concentration of 250 ng/μL and a Cas9 concentration of 250 ng/μL: the y1 + s4 treatment (125 ng/μL y1 and 125 ng/μL s4) (*p* = 0.0101) and the y2 treatment (250 ng/μL y2) (*p* = 0.0031).

The four CRISPR/Cas9 treatments did not differ significantly in the efficiency of adult transformation to a yellow knock‐out phenotype (χ^2^(3) = 2.83, *p* = 0.4181). However, the treatments did differ in how many individuals were in the category “100% yellow” (χ^2^(3) = 25.65, *p* < 0.001). Similar to larval knock‐out efficiency, the y1 + y3 treatment, with a sgRNA concentration of 500 ng/μL (250 ng/μL y1 and 250 ng/μL y3) and a Cas9 concentration of 500 ng/μL, was significantly more successful than the two treatments with a sgRNA concentration of 250 ng/μL and a Cas9 concentration of 250 ng/μL: the y1 + s4 treatment (125 ng/μL y1 and 125 ng/μL s4) (*p* < 0.001), and the y2 treatment (250 ng/μL y2) (*p* = 0.0181).

From each of the four treatments with a sgRNA/Cas9 complex, 3 larvae, scored as knock‐outs based on their head capsules, were followed to adult eclosion to assess the relationship between knock‐out head capsule and adult phenotype. Out of the 12 larvae in total, 9 survived to adulthood. Of these, 4 appeared 100% yellow, 4 were more than 50% yellow, and one appeared wildtype (Table 3). Therefore, the proportion of individuals with a knock‐out phenotype as both larvae and adults was approximately 0.89.

**TABLE 3:** Categorization of adult phenotype from larvae with knock‐out head capsules followed to eclosion.

Treatment	Concentration (ng/μL)	Larvae	Adults	Wildtype	< 50% yellow	> 50% yellow	100% yellow
y1 + 3/Cas9	250 + 250/500	3	2				2
y1 + s4/Cas9	125 + 125/250	3	1			1	
y1/Cas9	500/500	3	3			2	1
y2/Cas9	250/250	3	3	1		1	1

The treatments significantly differed in egg survival (χ^2^(5) = 81.47, *p* < 0.001). The non‐injected control had a significantly higher egg survival than each of the injected treatments (water‐injected control *p* < 0.001; y1 + y3 *p* < 0.001; y1 + s4 *p* < 0.001; y1 *p* < 0.001; y2 *p* < 0.001), and the water‐injected control additionally had higher egg survival than the y1 + s4 treatment (*p* = 0.0022). However, from egg hatching to adult eclosion, there was no significant difference in survival between the treatments (χ^2^(5) = 3.71, *p* = 0.5923) (Table 2).

### 3.2 Germline Generation

**FIGURE 5:**
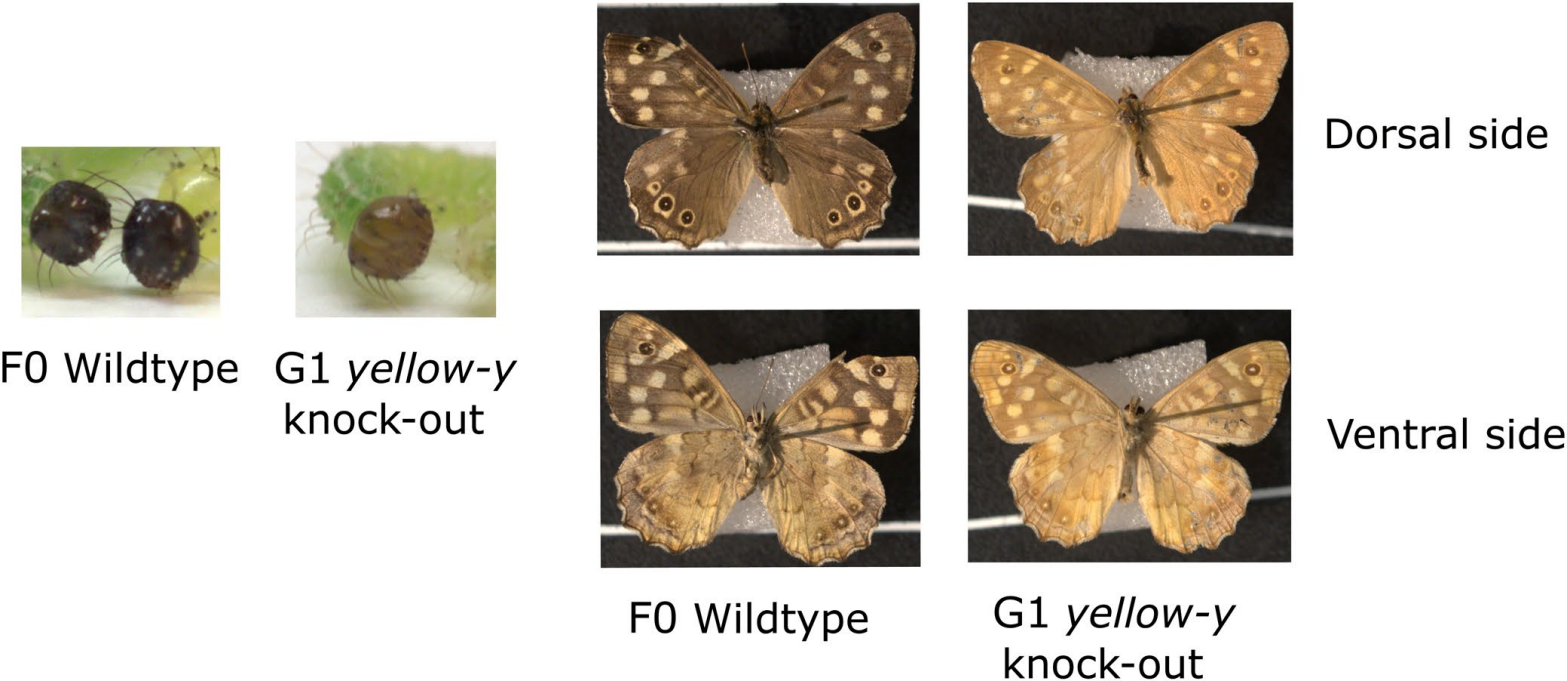
Pictures illustrating the phenotypic categories for larvae and adult *yellow‐y* knock‐outs in the G1. All the larvae had light brown first instar head capsules, indicating that the knocked‐out version of the *yellow‐y* gene had been inherited. Their head capsules can be compared to the dark brown wildtype head capsule. All the adults also appeared “100% yellow” in comparison to the wildtype background color.

Matings were performed between adults classified as “> 50% yellow” and “100% yellow” coming from three different treatments (y1 + y3, y1 + s4, and y1). In total, 75 larvae were phenotyped, and all of them where scored as knock‐outs. Of these, 59 larvae survived to adulthood, and all resulting adults appeared fully yellow (Table 4 and Figure 5).

**TABLE 4:** Germline transformations of larvae and adults from the two observed matings (all categorized as “100% yellow”), as well as unobserved matings (adults from categories “> 50% yellow” and “100% yellow”).

Parents (F × M)	Larvae	Knock‐out larvae	Survival to adult eclosion	100% yellow
Mating a (y1 + y3 × y1 + y3)	15	15	14	14
Mating b (y1 × y1 + y3)	15	15	13	13
Cage matings^a^	45	15	32	32

^a^ Cages with unobserved matings between adults from treatments y1 + y3, y1 + s4, and y1.

**FIGURE 6:**
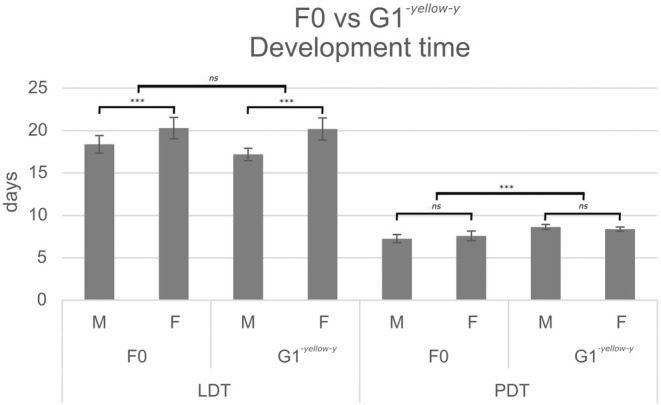
The mean and CI of the development times in days for the non‐injected wildtype control (F0) and germline transformed (G1‐*yellow‐y*) individuals, split by sex (M, male; F, female). The black lines indicate whether there was a significant difference between the two sexes or the two groups (F0 or G1‐*yellow‐y*). Significance levels were derived from the models LDT ~ Group + Sex and PDT ~ Group + Sex. LDT, larval development time; PDT, pupal development time.

**FIGURE 7:**
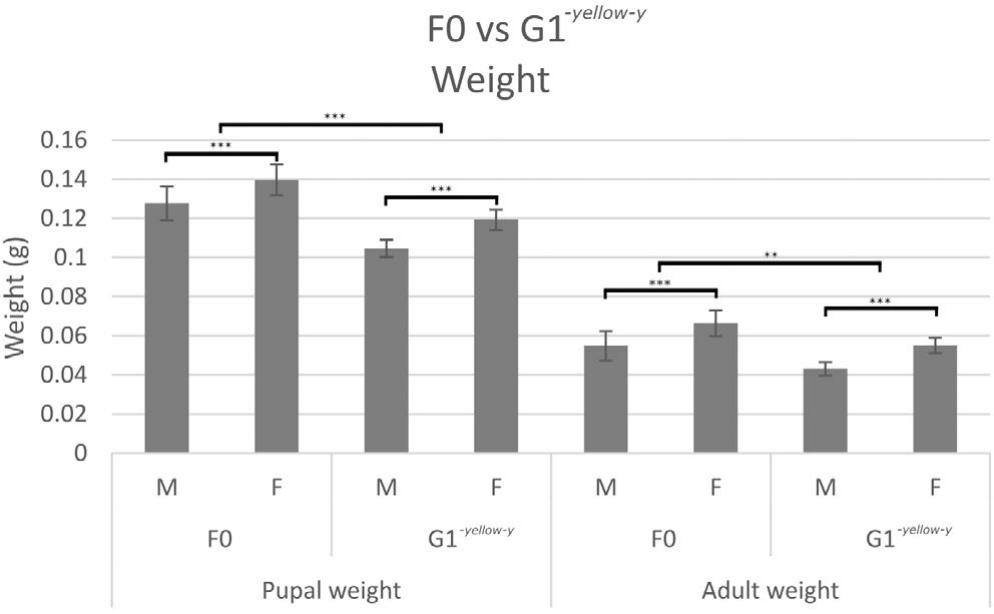
The mean and CI of the weights, in grams, of the non‐injected wildtype control (F0) and germline transformed (G1‐*yellow‐y*), split by sex (M, male; F, female). The black lines indicate whether there was a significant difference between the two sexes or the two groups (F0 or G1‐*yellow‐y*). Significance levels were derived from the models Pupal weight ~ Group + Sex and Adult weight ~ Group + Sex.

There was no significant difference in survival to adulthood between the germline *yellow‐y* gene knock‐outs (G1^‐*yellow‐y*^) and the non‐injected control (F0) (χ^2^(1) = 1.31, *p* = 0.2533). Similarly, there was no significant difference in larval development time (F_(1,39)_ = 0.22, *p* = 0.6401), although males developed significantly faster than females in both categories (F_(1,39)_ = 17.39, *p* < 0.001) (Figure 6). In some contrast, pupal development time was significantly longer for the germline transforms (F_(1,39)_ = 30.73, *p* < 0.001), while there was no significant difference between the sexes (F_(1,39)_ = 0.07, *p* = 0.796). Pupal weights of the germline transformants were significantly lower than those of the non‐injected controls (F_(1,39)_ = 37.15, *p* < 0.001), with males weighing significantly less than females in both categories (F_(1,39)_ = 18.86, *p* < 0.001) (Figure 7). Similarly, adults of the germline generation were significantly lighter in weight than the non‐injected control (F_(1,32)_ = 11.21, *p* = 0.0021), with males again weighing less than females (F_(1,32)_ = 21.49, *p* < 0.001).

## 4 Discussion

Here, we demonstrate that CRISPR/Cas9 is a highly efficient tool for inducing germline mutations in the ecological model species *P. aegeria*. Targeting the *yellow‐y* gene, more than 50% of the injected larvae exhibited knock‐out head capsules, and nearly 80% of surviving adults displayed at least partial transformation. Extending beyond the typical scope of CRISPR/Cas9 experiments, we generated a G1 generation by mating approximately 25 adults that were at least 50% transformed. All resulting offspring displayed full *yellow‐y* knock‐out phenotypes as both larvae and adults, suggesting that either all mated individuals carried homozygous germline mutations or that *yellow‐y* LOF mutations are dominant.

All four CRISPR/Cas9 treatments were successful and did not differ significantly in the proportion of adults displaying visible mutations. However, a significantly higher proportion of adults appeared 100% transformed in the treatment with the highest concentration of Cas9‐sgRNA in the complex (500 ng/μL sgRNA and 500 ng/μL Cas9) and two different sgRNAs targeting the *yellow‐y* gene. While it is not possible to separate the effects of sgRNA concentration and the number of sgRNAs in this study, the observed increase in effectiveness is in line with previous findings (Bassett et al. 2013; Wang et al. 2013). Increased overall efficiency as a result of higher Cas9‐sgRNA concentrations has previously been demonstrated in *Drosophila melanogaster* (Bassett et al. 2013). Similarly, in the silkworm ( *Bombyx mori* ), combining two sgRNAs significantly increased the proportion of highly transformed individuals when targeting the *BmBLOS2* gene, compared to the separate use of each sgRNA (Wang et al. 2013). However, it is important to note that using multiple sgRNAs can result in large deletions that extend across the targeted region (Mazo‐Vargas et al. 2022; Livraghi et al. 2024). This is especially important to consider when targeting non‐coding sequences or candidate genes that are closely linked to other genes.

Similar to other Lepidoptera (Perry et al. 2016; Liu, Han, et al. 2020; Liu, Zhang, et al. 2020; Shirai et al. 2021), the *yellow‐y* gene influences the background color of adult wings in *P. aegeria*. Here, we also report that the *yellow‐y* gene is involved in the melanization of the first instar head capsule. The correlation between individuals showing both the larval and adult knock‐out phenotypes was very high, suggesting they were due to the same genetic transformation. Unfortunately, it was not possible to conclusively determine if there were any strong pleiotropic effects on life history traits resulting from knocking‐out the *yellow‐y* gene. The germline‐transformed individuals and the non‐injected wildtype controls were reared at different time points, so while significant differences were found, the variation in life history traits was not much larger than what is typically observed between lines not reared in parallel (Aalberg Haugen et al. 2012). Further investigation, rearing germline‐transformed individuals alongside wildtype animals, is needed to distinguish between effects of the experimental design and any potential pleiotropic effects of the *yellow‐y* gene. Whether the *yellow‐y* gene influences growth and development may vary between species. For instance, this gene affects hatching success in the tobacco cutworm ( *Spodoptera litura* ) (Liu, Han, et al. 2020; Liu, Zhang, et al. 2020; Shirai et al. 2021), but not in the diamondback moth (*Plutella xylostella*) (Wang et al. 2021).

Microinjections were found to cause a lower hatching success. As there was no general difference between the injection of sgRNA/Cas9 complexes and the water‐injected control, this seemed to be due to the injection itself, possibly due to loss of egg yolk. Nevertheless, while microinjections may increase egg mortality, the hatching success achieved here (between 21% and 37%) is similar to results from other butterflies (Li et al. 2015; Matsuoka and Monteiro 2018). After hatching, survival until adult eclosion was not significantly affected by the treatments, consistent with observations in the black cutworm ( *Agrotis ipsilon* ) (Cao et al. 2018).

With the CRISPR/Cas9 technology effective and capable of inducing germline mutations in the ecological model species *P. aegeria*, it will be possible to directly manipulate candidate genes of physiological non‐visible traits that affect ecologically important traits. This includes traits where *P. aegeria* is already a well‐studied model organism, such as seasonal adaptation, voltinism, wing morphology, and male mating strategies (Van Dyck, Matthysen, et al. 1997; Van Dyck, Matthysen, et al. 1997; Wiklund and Persson 1983; Shreeve 1986; Nylin et al. 1989; Merckx and Van Dyck 2006; Aalberg Haugen and Gotthard 2015; Pruisscher et al. 2018; Lindestad et al. 2022). Within this context, reports of efficient CRISPR/Cas9 genome editing of circadian clock genes in the European corn borer moth, *Ostrinia nubilalis* , and the cotton bollworm, *Helicoverpa armigera*, similarly document a growing trend to begin exploring gene manipulations beyond morphological traits in Lepidoptera (Dayton et al. 2024; Jin et al. 2024). While a new target gene necessitates the design of new sgRNAs, the present study suggests that a total concentration of 500 ng/uL sgRNA and 500 ng/uL Cas9 in the complex, along with two different sgRNAs targeting a single gene, is more efficient than using 250 ng/uL sgRNA and 250 ng/uL Cas9 with a single sgRNA. By applying the present protocol to induce germline mutations, the CRISPR/Cas9 technology enables the exploration of the genetic foundations of non‐visible physiological traits in *P. aegeria*, bridging the gap between genotype variations, adaptive phenotypic changes, and ultimately, fitness (Bono et al. 2015).

## Author Contributions

**Anna B. Shoshan:** conceptualization (supporting), data curation (lead), formal analysis (lead), investigation (lead), methodology (supporting), project administration (equal), visualization (lead), writing – original draft (lead). **Kalle Tunström:** conceptualization (equal), investigation (supporting), methodology (equal), project administration (equal), supervision (supporting), validation (equal), writing – review and editing (supporting). **Christopher W. Wheat:** conceptualization (equal), methodology (equal), project administration (equal), resources (supporting), supervision (equal), validation (equal), writing – review and editing (equal). **Karl Gotthard:** conceptualization (equal), funding acquisition (lead), investigation (supporting), methodology (supporting), project administration (equal), resources (lead), supervision (lead), validation (equal), writing – review and editing (equal).

## Conflicts of Interest

The authors declare no conflicts of interest.

## Data Availability

The data supporting the findings of this study are available from the Dryad repository at https://doi.org/10.5061/dryad.9ghx3fftg.
